# Systematic review: risk prediction models for metachronous advanced colorectal neoplasia after polypectomy

**DOI:** 10.1111/jgh.16682

**Published:** 2024-07-30

**Authors:** James H‐E Kang, Emma Levine, Alex Fleet, Mc Stephen Padilla, Jeffrey K Lee, Hannah Harrison, Juliet A Usher‐Smith

**Affiliations:** ^1^ Department of Public Health and Primary Care University of Cambridge Cambridge UK; ^2^ University of California, San Francisco San Francisco California USA; ^3^ Kaiser Permanente Northern California Division of Research Oakland California USA

**Keywords:** cancer screening, colonoscopy, colorectal cancer

## Abstract

**Background and Aim:**

Colorectal cancer (CRC) is the fourth leading cause of cancer death globally. CRC surveillance is a common indication for colonoscopy, representing a considerable burden for endoscopy services. Accurate identification of high‐risk patients who would benefit from more intensive surveillance, as well as low‐risk patients suitable for less frequent follow‐up, could improve the effectiveness of surveillance protocols and resource use. Our aim was to identify and critically appraise published risk models for the occurrence of metachronous advanced colorectal neoplasia (ACN), defined here as CRC or advanced adenomas detected during surveillance colonoscopy.

**Methods:**

We searched PubMed and EMBASE for primary research studies reporting the development and/or validation of multivariable models that predict metachronous ACN risk. Screening of studies for inclusion, data extraction, and risk of bias assessment were conducted by two researchers independently.

**Results:**

We identified nine studies describing nine risk models. Six models were internally validated and two were externally validated. No model underwent both internal and external validation. Good model discrimination (concordance index > 0.7) was reported for two models during internal validation and for one model during external validation. Calibration was acceptable when assessed (*n* = 4). Methodological limitations and a high risk of bias were observed for all studies.

**Conclusions:**

Several published models predicting metachronous ACN risk showed some promise. However, adherence to methodological standards was limited, and only two models were externally validated. Head‐to‐head comparisons of existing models using populations independent from model development cohorts should be prioritized to identify models suitable for use in clinical practice.

## Introduction

Colorectal cancer (CRC) is the fourth most common cause of cancer death globally.^1^ Population‐based screening and surveillance with endoscopic removal of premalignant polyps significantly reduces both incidence and mortality.^2^ Post‐polypectomy surveillance represents a significant burden on endoscopy units, accounting for over 20% of colonoscopies performed in individuals aged ≥50 years.^3^

Societal guidelines recommend that polyps detected at a colonoscopy performed for any indication, screening or otherwise, should be resected. Surveillance colonoscopy to look for further incident advanced colorectal neoplasia (ACN) should then be offered to individuals who remain at higher‐than‐baseline risk of developing CRC despite polypectomy.^4^, ^5^ ACN is a composite term encompassing advanced colorectal polyps (“advanced adenomas”) and invasive CRC. In current guidelines, the need for, and frequency of, surveillance is determined by the presence and characteristics of polyps detected at the most recent colonoscopy, that is, polyp number, size, and histology.^4^, ^5^

However, the effectiveness of risk stratification based solely on previous colonoscopy findings is uncertain.^6^ A particular concern of current surveillance protocols is the under‐ or overutilization of colonoscopies in individuals with higher or lower risks of CRC, respectively.^7^ Since the majority of patients with colorectal polyps will not develop CRC,^8^ the risk of complications due to repeat procedures may not outweigh the benefits in lower risk individuals. Overutilization in this group also represents inefficient use of limited resources. By contrast, not surveying high risk individuals frequently enough may result in missed or delayed diagnoses.

Incorporating individual‐level CRC risk factors beyond baseline examination findings into risk assessments for post‐polypectomy ACN could increase the effectiveness and benefit–harm balance of surveillance. Multiple models have been developed to predict risk of metachronous ACN, defined here as ACN detected during surveillance colonoscopy,^9^ using different combinations of patient, polyp, and procedural risk factors. However, it is unclear which, if any, of these existing models make sufficiently accurate predictions of metachronous ACNs to be considered for use in clinical practice.

The aim of this systematic review is to identify and assess published risk models for metachronous ACN development following polypectomy. We will compare the identified models, in terms of their characteristics and performance, to determine those most suitable for implementation within risk‐stratified, personalized surveillance strategies.

## Methods

We conducted and report this study according to the Preferred Reporting Items for Systematic Reviews and Meta‐Analyses guidelines^10^ following an a priori protocol registered on PROSPERO (CRD42020205206).

### Search strategy

We performed a literature search of PubMed and EMBASE from inception to January 15, 2024 (see Data S1 for search strategy). Papers were limited to English‐language articles only. Additionally, we manually screened the reference lists of all included papers.

### Study selection

We included observational studies that developed and/or validated a model based on patient‐level data to predict metachronous ACN risk, provided they:

1. Were published as a primary research paper in a peer‐reviewed journal.
2. Identified risk factors for developing metachronous ACN at the level of the individual following a previous colonoscopy where polyps were identified.
3. Provided a measure of relative or absolute risk using a combination of two or more risk factors that allows identification of people at higher risk of metachronous ACN. This need not include more than one of the following categories: patient, polyp, and procedural risk factors.
4. Included a recognized performance metric (e.g. discrimination, calibration) or estimated impact on clinical decision‐making.

We excluded studies if:

1. Study populations consisted exclusively of specific groups of patients at higher risk of CRC: inflammatory bowel disease, genetic polyposis syndromes (e.g. Lynch syndrome), past history of CRC.
2. Basic science articles, review articles, and editorials.
3. Conference proceedings.

One reviewer (JH‐EK) performed the search. Two reviewers (JH‐EK and EL) screened the titles and abstracts to exclude papers that were clearly not relevant. Full‐text papers were examined when a definite decision to reject could not be made based on title and abstract alone. Two reviewers (JH‐EK and EL) independently assessed all full‐text papers. Those deemed not to meet inclusion criteria by both researchers were excluded. Disagreements were discussed until a consensus decision was made.

### Data extraction and quality assessment

Included studies were classified using the framework outlined in the “transparent reporting of a multivariable prediction model for individual prognosis or diagnosis” (TRIPOD) statement,^11^ according to whether they developed a new model, validated an existing model, or a combination of these. The CHecklist for critical Appraisal and data extraction for systematic Reviews of prediction Modelling Studies (CHARMS)^12^ was used to guide data extraction. The Prediction model Risk Of Bias ASsessment Tool (PROBAST), a series of signaling questions across four domains (participants, predictors, outcome, and analysis), was used to assess risk of bias and applicability of the models to risk‐stratified surveillance for ACN post‐polypectomy.^13^ Data were extracted independently by two researchers (JH‐EK, EL) using a standardized data collection tool based on the CHARMS and PROBAST checklists.^14^

### Model assessment

We primarily assessed predictive performance based on reported measurements of discrimination and calibration, although other metrics including sensitivity and specificity were also extracted. Discrimination is the extent to which predicted risks differentiate between patients with and without the outcome (metachronous ACN) in a specific cohort. It is measured by the area under the receiver operating characteristic curve (AUROC), which, for a binary outcome, is equivalent to the concordance index (C‐index/statistic). An AUROC/C‐index is 0.5 if prediction is no better than a random coin toss and 1.0 for a model that always assigns cases a higher score than controls. Consistent with existing methodological literature,^15^, ^16^, ^17^, ^18^ we defined discriminatory performance as poor for C‐indexes of 0.50–0.70, good for C‐indexes > 0.70–0.80, and excellent for C‐indexes ≥ 0.80.

Calibration is the extent to which the risk of an event predicted by a model corresponds to the observed frequencies of that event (as above, in a specific cohort). Measures of calibration extracted in this review included the Hosmer–Lemeshow (HL) test, observed‐to‐expected (O:E) risk ratios, and calibration plots.

## Results

### Literature search

**Figure 1:**
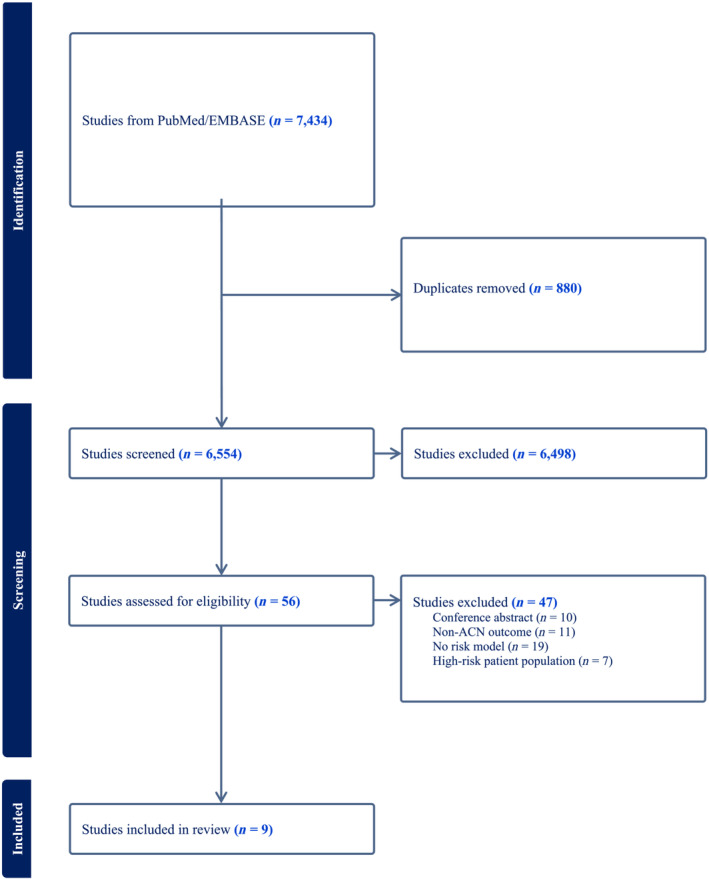
PRISMA flow diagram.

The literature search yielded 7434 articles. After de‐duplication, 6554 papers remained of which 6498 were excluded during title and abstract screening. Of the 56 remaining articles, 47 were excluded during full‐text screening. The most common reasons for exclusion at this stage were the absence of risk model (*n* = 19) and non‐ACN outcome (*n* = 11). Other reasons can be found in Figure 1.

### Model development

Table 1 summarizes the nine identified studies, each of which describe the development of a risk prediction model for metachronous ACN. The study populations used for model development were based in the United States (*n* = 5),^19^, ^20^, ^21^, ^22^, ^23^ Europe (*n* = 3),^24^, ^25^, ^26^ and South Korea (*n* = 1).^27^ Six studies used retrospective cohorts,^20^, ^23^, ^24^, ^25^, ^26^, ^27^ one prospective population‐based cohort data,^19^ one data from a previous randomized controlled trial,^22^ and the final study combined data from seven prospective studies including six intervention trials.^22^ Of the six retrospective cohorts: Three were single center^23^, ^24^, ^27^; the others used a multicenter study,^26^ a national screening database,^26^ and a national cancer registry.^25^ Study size ranged from 965 to 236 089 participants. All models were developed using populations containing both sexes, although one study conducted within the US‐based Veteran Affairs integrated healthcare system comprised almost exclusively (96.6%) male participants.^22^ Seven studies limited recruitment to participants with at least one adenoma^19^, ^20^, ^21^, ^22^, ^23^, ^25^ or, in one case, advanced adenoma^24^ at index colonoscopy. All studies excluded patient belonging to specific populations at higher baseline risk of CRC (e.g. inflammatory bowel disease, genetic polyposis syndromes) or who underwent incomplete colonoscopies. Seven models used metachronous ACN and two used incident metachronous CRC as the outcome^19^, ^20^, ^22^, ^23^, ^24^, ^25^, ^27^.^21^, ^26^ The risk index by Imperiale *et al*. was designed to stratify the risk of advanced adenoma at the second surveillance colonoscopy specifically.^23^

**Table 1:** Study characteristics

Author, year	Country	Study setting, population (*n*)	Enrollment period	Inclusion criteria	Exclusion criteria	Outcome (“event”) definition	Tripod level^†^ (validation method)
Imperiale 2014	USA	Retrospective cohort, single center *n* = 965	2005–2010	Adenoma at index colonoscopy that was performed for any reason except for surveillance of previous adenoma or cancer ≥2 surveillance exams	First‐degree relative diagnosed with CRC at <60 years, history of CRC, IBD, hereditary polyposis syndromes, colonoscopy subsequent to index exam for any reason other than surveillance	Metachronous ACN (AA or CRC) at second surveillance colonoscopy	3 (External, using independent dataset of 372 subjects from different medical center)
Facciorusso 2016	Italy	Retrospective cohort, single center *n* = 843	2004–2008	AA with complete resection and retrieval for pathology	History of CRC, familial polyposis syndromes, IBD, incomplete follow‐up data	Metachronous ACN (AA or CRC) at 3 years	1b (Internal, 10‐fold cross‐validation, repeated 250 times using bootstrapping)
Lee 2016	South Korea	Retrospective cohort, single screening center *n* = 11 042	2003–2011	Self‐referred for screening colonoscopy, asymptomatic, 40–70 years old age, surveillance exam at an interval of ≥2 years	History of colorectal surgery, IBD, incomplete exam to caecum or poor bowel prep, incomplete baseline questionnaire	Metachronous ACN (AA or CRC)	2a (Internal, random 70:30 split of data into development + validation datasets)
Liu 2016	USA	7 prospective cohorts (including 6 intervention trials) *n* = 8228	1984–1998	*Common to all constituent studies*: Baseline polypectomy and repeat surveillance exam within 3–5 years	*Common to all constituent studies*: History of CRC	Metachronous ACN within 3–5 years of polypectomy	2a (Internal, random 2:1 split of data into development + validation datasets)
Van Heijningen 2016	Netherlands	Retrospective cohort, cancer registry *n* = 2914	1988–2002	Aged ≥40 years, ≥1 adenoma	History of CRC, partial bowel resection, IBD, hereditary CRC syndromes. Missing pathology or colonoscopy report, poor bowel prep, colonoscopy reach no further than the distal colon	Metachronous ACN (AA or CRC)	1b (Internal, bootstrapping procedure with 1000 samples; cross‐validation)
Anderson 2018	USA	Randomized trial, multicenter *n* = 1948	2004–2008	Ages 45–75 years, with ≥1 adenoma resected (≥2 mm) within 4 months of study entry, with subsequent polyp‐free bowel and 3‐ or 5‐year surveillance	*Trial exclusion criteria*: History of IBD, CRC, familial CRC syndrome, bowel resection, specified comorbidities and medications Missing/incomplete pathology	Metachronous ACN	1a (Apparent performance only)
Wieszczy 2020	Poland	Retrospective cohort, national screening program *n* = 236 089	2000–2011	National screening programme participants: ages 50–66, asymptomatic, invited for colonoscopy every 10 years; people with family history from age 40	History of CRC, familial polyposis syndrome, incomplete exam due to failed cecal intubation, poor bowel prep, incomplete polyp resection, lack of histology	Incident CRC and fatal CRC	1b (Internal, bootstrapping procedure with 1000 samples)
Gupta 2022	USA	Retrospective cohort, multicenter *n* = 30 897	2004–2016	Baseline removal of adenoma or sessile serrated lesion; ≥1 surveillance colonoscopy >1 year after baseline exam	IBD, CRC ≤ 1 year of baseline exam, history of CRC, missing documentation of adequate bowel preparation or exam completion to cecum	ACN, defined as first occurrence of AA, incident CRC or fatal CRC ≥ 1 year after baseline colonoscopy	2a (Internal, random 2:1 split of data into development + validation datasets)
Knudsen 2023	USA	3 prospective population‐based cohorts: Nurses' Health Study (NHS) I/II; Health Professional Follow‐up Study (HPFS) *n* = 26 741	1986–2017 NHS I: 1986–2014 NHS II: 1991–2015 HPFS: 1986–2010	First‐time polypectomy NHS I: Female nurses aged 30–55 in 1976 NHS II: Female nurses aged 25–42 in 1989 HPFS: Male health aged 40–75 professionals in 1986	Death or CRC ≤ 6 months following baseline	CRC ≥ 6 months after polypectomy	3 (External validation study used electronic healthcare records from an integrated healthcare system (*n* = 76, 603 including 241 CRC cases) with an enrollment period of 2007–2018)

^†^ Types of prediction model studies for each model defined according to the TRIPOD guidelines: 1a, development only, performance assessed in training dataset only; 1b, development and validation using resampling; 2a, random split‐sample development and validation; 2b, nonrandom split‐sample development and validation; 3, development and validation using separate data; 4, validation study.

AA defined as adenoma ≥10 mm, with villous histology or high‐grade dysplasia.

AA, advanced adenoma; ACN, advanced colorectal neoplasia; CRC, colorectal cancer; IBD, inflammatory bowel disease.

Table 2 outlines the development of the nine models. Five were developed using logistic regression,^19^, ^20^, ^22^, ^23^, ^25^ and four used survival analyses (Cox proportional hazards regression).^21^, ^24^, ^26^, ^27^ Statistical predictor selection procedures were used to develop seven models: Three studies used backward or forward multivariable stepwise selection^21^, ^24^, ^26^; three combined regression‐based selection with bootstrapping procedures to examine the stability of the order of entry of each predictor^20^, ^22^, ^27^; one incorporated predictors based on multivariate associations in combination with Akaike's information criterion.^25^ Univariable variable–outcome associations informed the preselection of predictors before multivariable modeling in five studies.^20^, ^22^, ^24^, ^25^, ^27^ In contrast, Imperiale *et al*. did not specify the methods used for variable selection,^23^ while Anderson *et al*. prespecified inclusion of a novel metric (“adenoma bulk”).^19^ Individual‐level patient risk factors for CAN (e.g. age, smoking) and/or polyp characteristics were considered for inclusion in all models: One assessed patient‐risk factors only,^23^ one examined polyp factors only,^19^ and seven evaluated use of both.^20^, ^21^, ^22^, ^24^, ^25^, ^26^, ^27^ Only four additionally considered procedural factors such as adenoma detection rate or type of polyp resection.^20^, ^21^, ^24^, ^25^ None of the studies evaluated biochemical or genomic data. Across the nine studies, 32 variables were considered (Table 3).

**Table 2:** Development of risk models

Author, year	Modeling method	Model variable selection method	Number of events	Candidate predictors considered for inclusion in final model	Predictors included in final model	EPV
Imperiale 2014	Logistic regression	Not reported	53	*Patient*: Age (categorical), sex, previous colonoscopy (AA *vs* non‐AA)	*Patient*: Age (categorical), sex, previous colonoscopy (AA *vs* non‐AA)	18
Facciorusso 2016	Cox regression	Univariable predictor–outcome associations, then multivariable stepwise backward selection	229	*Patient*: Age (dichotomous), gender *Polyp*: Size (dichotomous), number (continuous), morphology (pedunculated *vs* sessile or non‐polypoid), location, villous/tubulovillous histology, grade of dysplasia *Procedural*: Type of resection (reference *en bloc*)	*Polyp*: Grade of dysplasia, size (dichotomous), number (dichotomous)	25
Lee 2016	Cox regression	Univariable predictor–outcome associations; multivariable stepwise backward selection with bootstrapping: Variable included if it appeared in >500/1000 resample models	150	*Patient*: Age (categorical), gender, smoking, alcohol consumption (categorical), BMI (categorical), family history of CRC, metabolic syndrome *Polyp*: ≥1 cm sessile serrated lesion, sessile serrated lesion, high‐risk versus low‐risk colorectal neoplasia (high risk defined as ACN or ≥3 adenomas; low risk defined as 1–2 tubular adenomas <10 mm)	*Patient*: Age (categorical), gender *Polyp*: Sessile serrated lesion, high‐risk versus low‐risk colorectal neoplasia	15
Liu 2016	Logistic regression	Univariable and multivariable predictor–outcome associations, then LASSO with 1000 bootstrap samples, Bayesian model averaging	NR	*Patient*: Age (continuous), BMI (continuous), sex, race/ethnicity, family history of CRC, smoking, history of polyps *Polyp*: Number (continuous), size (continuous), location, villous/tubulovillous histology, grade	*Patient*: Age (continuous), history of polyp *Polyp*: Number (continuous), size (continuous), tubulovillous/villous histology, location	N/A
Van Heijningen 2016	Logistic regression	Univariable, then multivariable predictor–outcome associations; Akaike's Information Criterion used to choose best fitting model	189	*Patient*: Age (continuous), sex *Polyp*: Size (dichotomous), number (categorical), location, villous histology, HGD *Procedural*: Surveillance interval, negative surveillance colonoscopy number	*Patient*: Age (continuous), sex *Polyp*: Size (dichotomous), number (categorical), location, villous histology *Procedural*: Surveillance interval (continuous), number of surveillance colonoscopies	21
Anderson 2018	Logistic regression	Pre‐specified	187	*Polyp*: “Adenoma bulk,” defined as the sum of diameters of all adenomas at index colonoscopy	*Polyp*: “Adenoma bulk” (dichotomous, <10 *vs* ≥10 mm)	187
Wieszczy 2020	Cox regression	Forward stepwise selection with recursive partitioning	439	*Patient*: Age (dichotomous), sex, family history of CRC *Polyp*: Size (categorical), number (dichotomous), villous/tubulovillous histology, grade of dysplasia	*Patient*: Age (dichotomous) *Polyp*: Adenoma size (dichotomous), grade of dysplasia	63
Gupta 2022	Logistic regression	Univariable and multivariable predictor–outcome associations, then LASSO with 1000 bootstrap samples, Bayesian model averaging	2046	*Patient*: Age (continuous), sex, race/ethnicity, BMI, diabetes, aspirin, smoking *Polyp*: Number (continuous), number (categorical), size (dichotomous), location, ≥1 non‐AA, ≥1 AA, villous/tubulovillous histology, HGD, large serrated polyp, hyperplastic polyp ≥10 mm, combination of AA/SSL *Procedural*: ADR (categorical)	*Patient*: Age (continuous), sex, diabetes, smoking, *Polyp*: Number (categorical), polyp location, size (dichotomous), villous/tubulovillous histology *Procedural*: ADR	108
Knudsen 2023^†^	Cox regression	Backward stepwise regression	220	*Patient*: Age (categorical), sex, ethnicity, family history of CRC, BMI (categorical), alcohol use (categorical), smoking, regular aspirin/NSAIDs, physical activity level (categorical) *Polyp*: Histology (tubular, serrated, tubulovillous, villous, carcinoma‐in‐situ, high grade dysplasia), number (dichotomous), size (dichotomous), location, ≥3 serrated polyps, ≥10 mm serrated polyp, serrated polyp location *Procedural*: ≥1 endoscopy prior to polypectomy, indication for colonoscopy	*Patient*: Age (categorical), sex, BMI (categorical), alcohol use (categorical) Polyp: Histology (tubular, serrated, tubulovillous, villous, carcinoma in situ, high‐grade dysplasia), number (dichotomous), size (dichotomous), serrated polyp location *Procedural*: ≥1 endoscopy prior to polypectomy	12

^†^ Knudsen *et al*. constructed models using different combinations of variables in their development and validation cohorts. We report on what the authors refer to as the “Final Model” in the development cohort.

AA defined as adenoma ≥10 mm, with villous histology or high‐grade dysplasia.

AA, advanced adenoma; ACN, advanced colorectal neoplasia; ADR, adenoma detection rate; BMI, body mass index; CRC, colorectal cancer; EPV, events per variable; HGD, high‐grade dysplasia; N/A, not applicable; NR, not reported; SSL, sessile serrated lesion.

**Table 3:** Thirty‐two candidate predictors assessed for inclusion in models across all studies

Patient	Age^†^ Sex^†^ Race/ethnicity Family history of CRC History of polyps^†^ History of AA^†^ Smoking^†^ Alcohol consumption^†^ Aspirin/NSAID use Physical activity level BMI^†^ Family history of CRC Diabetes/metabolic syndrome^†^
Polyp	Adenoma size^†^ Adenoma number^†^ Adenoma morphology (pedunculated, sessile, non‐polypoid) Colonic location^†^ Histology (tubular, tubulovillous, villous, serrated)^†^ Grade of dysplasia^†^ Presence of SSL^†^ SSL number SSL size Combination of AA/SSL High vs low risk classification as per USMSTF guidelines (high risk defined as ACN or ≥3 adenomas; low risk defined as 1–2 adenomas <10 mm)^†^ Hyperplastic polyp ≥10 mm “Adenoma bulk,” defined as the sum of diameters of all adenomas at index colonoscopy^†^
Procedural	Adenoma detection rate^†^ Indication for colonoscopy Type of resection (en bloc, piecemeal) Surveillance interval^†^ Negative surveillance colonoscopy number History of ≥1 endoscopy prior to polypectomy^†^

^†^ Included in the final risk prediction model in at least one of the reviewed studies.

Knudsen *et al*. constructed models using different combinations of variables in their development and validation cohorts. We report on what the authors refer to as the “Final Model” in the development cohort.

AA, advanced adenoma; ACN, advanced colorectal neoplasia; BMI, body mass index; CRC, colorectal cancer; SSL, sessile serrated lesion.

### Model validation

Six studies included some form of internal validation.^20^, ^22^, ^24^, ^25^, ^26^, ^27^ Three used *n*‐fold cross‐validation or bootstrapping procedures (TRIPOD level 1b).^24^, ^25^, ^26^ The other three studies randomly split the original cohort into development and validation datasets (TRIPOD level 2a).^20^, ^22^, ^27^ Two studies carried out external validations to evaluate model performance in datasets distinct from the development population (TRIPOD level 3). Specifically, Imperiale *et al*. used distinct hospital‐based cohorts for model development and validation.^23^ Knudsen *et al*. developed their model using three US population‐based cohorts (Nurses' Health Study I/II and Health Professionals Follow‐up Study) before conducting validation studies in an electronic health record based clinical cohort of individuals who had undergone colonoscopy in an integrated healthcare system.^21^

### Model performance

Table 4 details the performance metrics measured for the nine models in development, internal validations, and external validations. Calibration was assessed in four studies^20^, ^22^, ^25^, ^27^: Three used the HL test to demonstrate good fit during model development^20^, ^22^, ^27^ with two of these also using the HL test in their internal validation.^22^, ^27^ By contrast, Gupta *et al*. did not report model calibration in validation data.^20^ Van Heijningen *et al*. reported observed vs predicted metachronous ACN frequencies across four cross‐validation samples yielding O:E ratios ranging from 0.76 to 1.41.^25^ Notably, calibration was not reported in either external validation study.^21^, ^23^ Model discrimination was measured in eight of nine model development cohorts and in six of seven validation cohorts. C‐index values ranged from 0.59 to 0.81 during model development^19^, ^20^, ^21^, ^22^, ^24^, ^25^, ^26^, ^27^ and 0.62 to 0.79 in internal validation data.^20^, ^22^, ^24^, ^25^, ^27^ Knudsen *et al*. reported a C‐index of 0.75 for their model during external validation.^21^ Imperiale *et al*. evaluated the performance of their risk index by examining the proportions of individuals classified as high and low risk who were subsequently diagnosed with ACN at the second surveillance colonoscopy.^23^ Subjects classified as low risk by the risk index had a 4.8% predicted risk of ACN at the second surveillance colonoscopy, whereas high‐risk individuals had 14.9% risk. With US Multisociety Task Force (USMSTF) guideline‐based risk stratification, the low‐risk subgroup had 4.5% predicted risk of metachronous ACN compared to 7.2% for high‐risk participants.^23^

**Table 4:** Risk model performance

		Apparent performance in development data			Validation dataset performance		
Author, year	Outcome	Calibration	Discrimination C‐index (95% CI)	Other measures/notes	Calibration	Discrimination C‐index (95% CI)	Other measures/notes
Imperiale 2014	ACN	NR	NR	Risk score at index colonoscopy resulted in more extreme absolute ACN risk predictions, compared to using AA versus non‐AA alone (as per USMSTF guidance). Authors suggest that this might aid clinician decision‐making about time interval between follow‐up exams	NR	NR	Risk score resulted in similar predicted absolute risks of ACN compared to derivation cohort
Facciorusso 2016	ACN	NR	0.81 (0.72–0.86)	Classification error rate: 0.09	NR	0.79 (0.72–0.83)	Classification error rate: 0.12
Lee 2016	ACN	HL *P* = 0.49	0.71 (0.66–0.76)	Number of surveillance colonoscopies needed to detect 1 metachronous ACN was lower when using score based on model compared to USMSTF guidelines	HL *P* = 0.75	0.65 (0.56–0.74)	Number of surveillance colonoscopies needed to detect 1 metachronous ACN was lower when using score based on model compared to USMSTF guidelines
Liu 2016	ACN	HL *P* = 0.39	0.68 (0.66–0.70)	*Model cutoff to improve sensitivity by 10% over 2012 USMSTF guidance*: Sens (95% CI): 0.88 Spec (95% CI): NR *Model cutoff to improve specificity by 10% over 2012 USMSTF guidance*: Sens (95% CI): NR Spec (95% CI): 0.51	HL *P* = 0.21	0.65 (0.62–0.69)	*Model cutoff to improve sensitivity by 10% over 2012 USMSTF guidance*: Sens (95% CI): 0.89 Spec (95% CI): 0.28 *Model cutoff to improve specificity by 10% over 2012 USMSTF guidance*: Sens (95% CI): 0.76 Spec (95% CI): 0.0.46
Van Heijningen 2016	ACN	NR	0.72 (0.69–0.76)	N/A	NR for full model; observed and expected risks reported for cross validation samples only	Full model: 0.71 (0.67–0.74)	*Discrimination*: c‐statistics compared between full model containing all considered predictors; model containing only polyp‐related factors; models based on Dutch, US, and UK surveillance guidelines *Calibration*: Predicted versus observed ACN risk reported for cross‐validation samples, giving observed‐expected risk ratios ranging from 0.76 to 1.41
Anderson 2018	ACN	NR	0.59	Sens: 0.40 Spec: 0.73	N/A	N/A	N/A
Wieszczy 2020	CRC	NR	0.63	2012 USMSTF C‐index: 0.627	NR	NR	Bootstrapping procedure performed to confirm the significance of risk factors selected to be in novel risk stratification system. Internal validation performed but associated performance metrics not reported
Gupta 2022	ACN	HL *P* = 0.24	0.63 (0.62–0.65)	*Model cutoff to optimize sensitivity*: Sens (95% CI): 0.69 (0.68–0.69) Spec (95% CI): 0.51 (0.48–0.53) *Model cutoff to optimize specificity*: Sens (95% CI): 0.57 (0.54–0.59) Spec (95% CI): 0.63 (0.63–0.64)	NR	0.62 (0.60–0.64)	*Model cutoff to optimize sensitivity*; Sens (95% CI): 0.66 (0.63–0.69) Spec (95% CI): 0.50 (0.49–0.51) NRI^†^: 10.6% correctly + 6.63% incorrectly reclassified as high risk *Model cutoff to optimize specificity*: Sens (95% CI): 0.55 (0.51–0.58) Spec (95% CI): 0.63 (0.62–0.64) NRI: 0.9% incorrectly reclassified as high risk; 6.4% correctly reclassified as low risk
Knudsen 2023^‡^	CRC	NR	0·71 (0·65–0·77)	N/A	NR	0·75 (0·70, 0·79)	The validation dataset model used the same predictors as the development dataset model, except for alcohol consumption that was unavailable NRI^†^ was determined using a separate model for the validation cohort that also included quality of bowel preparation and cecal intubation as predictors: 13% total cohort reclassified, although it was not specified what proportion were correctly versus incorrectly reclassified

^†^ Net reclassification improvement: USMTSF 2020 guidelines used as reference of comparison.

^‡^ Knudsen *et al*. constructed models using different combinations of variables in their development and validation cohorts. We report on what the authors refer to as the “Final Model” in the development cohort.

AA defined as adenoma ≥10 mm, with villous histology or high‐grade dysplasia.

AA, advanced adenoma; ACN, advanced colorectal neoplasia; C‐index, concordance index; CRC, colorectal cancer; HL, Hosmer–Lemeshow statistic; NR, not reported; NRI, net reclassification index; sens, sensitivity; spec, specificity; USMSTF, US Multisociety Task Force.

Seven studies compared model predictive performance to established guidelines. The discrimination of four models was shown to be comparable to existing surveillance protocols.^19^, ^21^, ^25^, ^26^ In the case of Van Heijningen *et al*., discriminatory performance in validation data was better for the novel model that incorporated patient, polyp, and procedural variables (C‐index: 0.707) compared to existing US, UK, and Dutch surveillance guidelines that used only polyp characteristics (C‐index: 0.664 for the US 2012 guideline, C‐index: 0.674 for the UK 2010 guideline, and C‐index: 0.642 for the Dutch 2002 guideline).^25^ Liu *et al*. and Gupta *et al*. considered the effect of choosing model cutoff values a priori to ensure 10% higher sensitivity compared to the USMSTF post‐polypectomy surveillance guidelines on specificity, and vice versa.^20^, ^22^ In both studies, prespecifying improvements sensitivity or specificity did not result in improvement in the other metric. Specifically, choosing cutoffs to ensure 10% improvement in specificity versus USMSTF guidelines did not improve sensitivity, while prespecifying cutoffs to achieve 10% improvement in sensitivity versus USMSTF guidelines actually reduced specificity.

Beyond predictive accuracy, several studies also considered the impact that model‐informed surveillance could have on colonoscopy resource use. Defining overuse as the proportion of individuals classified as high risk who do not go on to develop ACN (i.e. 1‐positive predictive value) and underuse as the proportion of those classified as low risk who later develop ACN (1‐negative predictive value), both Liu *et al*. and Gupta *et al*. estimated that use of model‐informed surveillance would result in comparable rates of colonoscopy over‐ and underuse to when USMSTF protocols are applied.^20^, ^22^ Similarly, although Wiesczczy *et al*.'s model did not exhibit superior discriminatory performance versus USMSTF guidelines (C‐index 0.626 *vs* 0.627), it classified 74% fewer patients as being high risk.^26^ This suggests that use of this model would result in fewer patients being recommended for surveillance at shorter intervals, thereby reducing colonoscopy burden.

### Model presentation

Four studies developed simplified scoring systems based on the model regression formulae.^23^, ^24^, ^25^, ^27^ Five studies provided coefficients for all risk factors included in their models.^20^, ^21^, ^24^, ^26^, ^27^ However, only Van Heijningen *et al*. shared their full model regression formula, that is, coefficients, and intercept corresponding to baseline ACN risk.^25^

### Risk of bias

Table 5 summarizes the risk of bias and applicability assessment for the included studies using the PROBAST framework^13^ with detailed information outlining the authors assessment contained in Tables S1a and S1b. No study met PROBAST standards for low risk of bias, that is, a low rating in all four domains. All models were assessed as low risk of bias in the “Participants,” “Predictors,” and “Outcomes” domains. However, concerns were raised in the “Analysis” domain for all studies: the most common indicators of bias related to the categorization of continuous predictor variables (*n* = 8) and the handling of missing data (*n* = 6).^11^

**Table 5:** Risk of bias and applicability assessment

Author, year	Risk of bias				Applicability			Overall	
	Participants	Predictors	Outcome	Analysis	Participants	Predictors	Outcome	**Risk of bias**	**Applicability**
Imperiale, 2014	+	+	+	−	−	+	−	−	−
Facciorusso, 2016	+	+	+	−	−	+	+	−	−
Lee, 2016	+	+	+	−	+	+	+	−	+
Liu, 2016	+	+	+	−	−	+	+	−	−
Van Heijningen, 2016	+	+	+	−	+	+	+	−	+
Anderson, 2018	+	+	+	−	−	+	+	−	−
Wieszczy, 2020	+	+	+	−	+	+	+	−	+
Gupta, 2022	+	+	+	−	−	+	+	−	−
Knudsen 2023	+	+	+	−	+	+	+	−	+

Green +: Low risk of bias/high applicability.

Red −: High risk of bias/low applicability.

Applicability is the extent to which study findings apply to the review question. The present review is concerned with models for predicting metachronous ACN risk in patients who undergo polypectomy, but do not belong to a subpopulation known to be at higher risk of CRC for whom dedicated surveillance protocols exist (e.g. hereditary cancer syndromes, inflammatory bowel disease). Five models were developed using data from or for participants that are not representative of the “general” ACN surveillance population of interest. Imperiale *et al*.'s risk index was designed exclusively for use after the first surveillance colonoscopy and before the second,^23^ limiting the opportunities in which it could be used in a clinical setting. Facciorusso *et al*.'s model was designed for use exclusively in individuals with AAs at baseline colonoscopy, so it would not be applicable to patients with only low‐risk adenomas.^24^ Anderson *et al*.'s and Liu *et al*.'s models were developed with data from clinical trials that had stringent exclusion criteria including use of lipid‐lowering medications or a diagnosis of osteoporosis.^28^, ^29^ Gupta *et al*. developed their model using an almost exclusively (96.6%) male cohort.^22^

## Discussion

### Principal findings

To our knowledge, this is the first systematic review of risk prediction models for postpolypectomy metachronous ACN. Nine models were identified, of which six have been internally validated and two externally validated. Discriminatory performance in validation ranged from poor (C‐index: 0.62) to good (C‐index: 0.79). Calibration was measured in three internal validations but in neither of the two external validations. High risk of bias was present in all studies, and applicability was limited for five models developed using populations not representative of most patients undergoing colonoscopic surveillance.

### Implications for future research

The models identified in this review show some potential to improve colonoscopic surveillance strategies by stratifying patients according to risk of developing metachronous ACN. For example, the model by Knudsen *et al*. exhibited good discriminatory ability for predicting CRC following polypectomy (C‐index: 0.71), has been externally validated (C‐index: 0.75), and enables calculation of risk using widely available predictors.^21^ Liu *et al*. and Gupta *et al*.^20^, ^22^ further demonstrated how the use of a single model could be adapted by setting different risk score cutoffs to achieve different aims: a cut‐off specified for high sensitivity and low specificity could be used to identify individuals at lower risk of metachronous ACN whose surveillance interval could be extended. By contrast, a high sensitivity and high specificity cutoff could define higher risk patients for more urgent examination.

An advantage of using models compared to current surveillance protocols is that, depending on which is chosen, different combinations of patient, polyp, and endoscopic procedural factors could be used for risk stratification. Specifically, all models incorporated predictors that are readily available in everyday clinical practice (Table 3), with the exception of the model by Anderson *et al*. that included a novel metric (“adenoma bulk”).^24^ However, this measure, comprising the sum of the estimated diameters of resected adenomas, could be easily obtained at the time of colonoscopy. Furthermore, four models were adapted into simplified risk scores that could be easily incorporated into clinical practice.^23^, ^24^, ^25^, ^27^

There is, however, an urgent need for external validation studies. Except for models by Knudsen *et al*. and Imperiale *et al*.,^21^, ^23^ no models were evaluated in patient populations entirely separate from training data. Therefore, an important next step will be to compare the existing models head‐to‐head in independent datasets ideally by independent investigators using standardized performance metrics of discrimination and calibration. This approach will help to identify the best performing models that can then be evaluated in impact studies before potential implementation in a real‐world setting.

In addition to validating existing models, there may be also be benefit in updating or developing additional ones. Some models were devised using older data, for example, enrollment took place entirely before 2000 in one study,^22^ and only two recruited patients after 2011.^20^, ^21^ Risk estimates based on historical endoscopy records might differ from more contemporary data due to enhanced tumor detection resulting from advancements in technology and endoscopist training. Updating or developing new models would also provide opportunities to include important CRC risk predictors not evaluated in the present studies that might enhance model performance. For example, endoscopist adenoma detection rate, an important prognostic factor for metachronous ACN,^20^, ^30^ was included in only one model.^20^ Colonoscopy quality performance metrics are emphasized in societal guidelines^31^ and measured routinely in clinical practice, so these should be considered as candidate predictor variables in future modeling studies. Biomarkers (e.g. fecal immunochemical test) are also increasingly commonplace and may improve predictive performance while maintaining applicability. Genetic‐based risk stratification is also of growing interest for CRC prevention.^32^, ^33^ Although population‐wide genomics are currently unavailable, advancements in genetic testing including reduced costs may soon make possible precise assessment of an individual's genetic risk.^34^ Finally, all studies in this review had a high risk of bias, mostly due to data handling issues in model development. Therefore, adherence to best practice methodological guidelines should be prioritized by investigators to ensure new models have maximum internal validity and potential for usefulness in clinical practice.

### Strengths and limitations

The main strengths of this review include its rigorous screening of studies for inclusion, data extraction, and quality assessment. Study selection was performed independently by two authors using an a priori protocol. Data extraction and risk of bias assessment was completed by four investigators using a standardized data collection tool.^14^ PROBAST, a quality assessment tool for risk prediction models,^13^ was used to perform a robust risk of bias assessment for each model and identify methodological limitations in included studies.

A limitation is that we were unable to exclude publication bias. Also, we could not exclude the existence of other published models indexed in alternative databases. However, we consider this to be unlikely since we manually searched the references of included studies. We did not review models developed using individuals at higher risk of ACN, so our findings will not be applicable to those with familial CRC syndromes, inflammatory bowel disease, previous cancer, or bowel resection. However, more intensive surveillance protocols already exist for these high‐risk groups. Most studies utilized European or US‐based cohorts, with one model developed in South Korea. No risk models were developed or validated in African, Arabic, or Australasian cohorts. CRC incidence varies up to 10‐fold according to nationality.^35^ Therefore, the models presented here may be less applicable to unrepresented geographical populations.

### Conclusion

We identified nine studies describing novel risk prediction models for metachronous ACN. Six models had undergone internal validation but only two had been externally validated. Three models exhibited good discrimination (C‐index > 0.7) in validation data, and calibration, when assessed, was satisfactory. Most models contained predictor variables that are readily available in current clinical practice. However, all studies exhibited methodological limitations and a high risk of bias when evaluated through the lens of best practice guidelines. Future investigations should focus on adherence to expert methodological standards and comparison of multiple existing models in independent datasets.

## Guarantor of article

James H‐E Kang.

## Supporting information


**Data S1.** Supporting Information.

**Table S1a:** CHARMS checklist. Relevant items to extract from individual studies for purposes of description and assessment of risk of bias and applicability. (Adapted from Moons KG. 2014).

**Table S1b:** PROBAST checklist. Prediction model study Risk of Bias Assessment Tool.
