# Supplementary material for: Systematic review: risk prediction models for metachronous advanced colorectal neoplasia after polypectomy
Source: J Gastroenterol Hepatol. 2024 Jul 30;39(12):2533–44. doi: 10.1111/jgh.16682 (PMC11660205; doi:10.1111/jgh.16682)
Supplement: Supplementary file 1 — Data S1. Supporting Information. [file JGH-39-2533-s002.docx]

PubMed search:

(colorectal neoplasms[mesh] OR colonic polyps[mesh] OR "advanced colorectal polyp" OR ("colorectal neoplasm"[Title/Abstract:~3]) OR ("colorectal cancer"[Title/Abstract:~3]))

AND

(“post-polypectomy” OR metachronous neoplasm[mesh] OR “metachronous” OR (“follow up second”[Title/Abstract:~5]) OR colonoscopy[mesh] AND (time factors[mesh] OR “second” OR “follow up” OR “lapsed time”))

AND

((Validate OR validation OR Predict[Title] OR Rule OR rules) OR ((Predict OR Prediction) AND (Outcome OR Outcomes OR Risk OR Risks OR Model OR Models OR Modelling)) OR ((History OR Variable OR Variables OR Criteria OR Score OR Scoring OR Scores OR Characteristic OR Characteristics OR Finding OR Findings OR Factor OR Factors) AND (Predict OR Prediction OR Model OR Models OR Modelling OR Decision OR Identify OR Identifying OR Prognostic OR Prognosis)) OR (Decision AND (Model OR Models OR Modelling OR Clinical OR Logistic)) OR (Prognostic AND (History OR Variable OR Variables OR Criteria OR Score OR Characteristic OR Characteristics OR Finding OR Findings OR Factor OR Factors OR Model OR Models OR Modelling)))

Embase search:

('colorectal tumor'/exp OR 'colon polyp'/exp OR "advanced colorectal polyp" OR (colorectal NEAR/3 neoplasm) OR (colorectal NEAR/3 cancer))

AND

(“post-polypectomy” OR ‘second primary neoplasm’/exp OR “metachronous” OR (‘follow up’ NEAR/5 second) OR ‘colonoscopy’/exp AND (‘time factor’/exp OR “second” OR “follow up” OR “lapsed time”))

AND

((Validate OR validation OR Predict:ti OR Rule OR rules) OR ((Predict OR Prediction) AND (Outcome OR Outcomes OR Risk OR Risks OR Model OR Models OR Modelling)) OR ((History OR Variable OR Variables OR Criteria OR Score OR Scoring OR Scores OR Characteristic OR Characteristics OR Finding OR Findings OR Factor OR Factors) AND (Predict OR Prediction OR Model OR Models OR Modelling OR Decision OR Identify OR Identifying OR Prognostic OR Prognosis)) OR (Decision AND (Model OR Models OR Modelling OR Clinical OR Logistic)) OR (Prognostic AND (History OR Variable OR Variables OR Criteria OR Score OR Characteristic OR Characteristics OR Finding OR Findings OR Factor OR Factors OR Model OR Models OR Modelling)))
