# Supplementary material for: Systematic review: risk prediction models for metachronous advanced colorectal neoplasia after polypectomy
Source: J Gastroenterol Hepatol. 2024 Jul 30;39(12):2533–44. doi: 10.1111/jgh.16682 (PMC11660205; doi:10.1111/jgh.16682)
Supplement: Supplementary file 2 — Table S1a: CHARMS checklist. Relevant items to extract from individual studies for purposes of description and assessment of risk of bias and applicability. (Adapted from Moons KG. 2014). [file JGH-39-2533-s001.pdf]

**SUPPLEMENTARY TABLE 1a: CHARMS checklist. Relevant items to extract from individual studies for purposes of description and assessment of risk of bias and applicability. (Adapted from Moons KG. 2014)**

| Domain/Key Items | Imperiale, 2014 | van Heijningen, 2015 | Facciorusso, 2016 | Lee, 2016 | Liu, 2016 | Anderson, 2018 | Wieszczy, 2020 | Gupta, 2022 | Knudsen, 2023 |
|---|---|---|---|---|---|---|---|---|---|
| **1. Source of data** | | | | | | | | | |
| Source of data | Retrospective cohort | Existing registry | Retrospective cohort | Retrospective cohort | Combined data (e.g. IPD of combination of cohorts) | Randomized trial | Existing registry | Retrospective cohort | Prospective cohort |
| **2. Participants** | | | | | | | | | |
| Recruitment method | Retrospective cohort | Retrospective recruitment | Retrospective recruitment | Retrospective recruitment | Data collected from 7 prospective studies including 6 x RCT | Prospective recruitment | All individuals meeting eligibility criteria between recruitment dates | Retrospective recruitment | Prospective cohort |
| Recruitment dates | 2005-2010 | 1988-2002 | 2004-2008 | 2003-2011 | 1984-1998 | 2004-2008 | 2000-2011 | 2004-2016 | 1986-2017 |
| Study setting | Single-center study | Nationwide histopathology registry | Single-center study | Single-center study | Prospective intervention study data | Vitamin D/Calcium Polyp Prevention Study | National Screening Programme database | Veterans Affairs Integrated Healthcare System | Population-based |
| Study sites (regions) | USA | Netherlands | Italy | South Korea | USA | USA | Poland | USA | USA |
| Study sites (number of centers) | 1 | 10 | 1 | 1 | Unclear - some studies were likely used in more than one study | 11 | 132 | 73 | 3 |
| Criteria inclusion | History of neoplastic polyp at index colonoscopy, performed for any indication except for surveillance of previous polyps or CRC. Need to have undergone 2 surveillance exams | Aged ≥40 years, ≥1 adenoma | Advanced colorectal adenoma (≥10 mm, villous component and HGD) at index colonoscopy with complete resection and retrieval for pathological analysis) | Age 40-70 years, asymptomatic, self-referred for screening colonoscopy, received a surveillance colonoscopy at >2 years | Other | Age 45-75 years, ≥1 x large adenoma (≥2 mm) histologically confirmed and removed 4 months prior to study, complete colonoscopy free of polyps, 3 or 5 years | Polish National Screening Programme participants: people aged 50-66 with no symptoms of CRC invited for colonoscopy every 10 years; individuals with FMHx start at age 40 | Individuals with colonoscopy and baseline removal of an adenoma or SSL, who also had at least 1 follow-up surveillance colonoscopy >1 year after baseline | First-time polypectomy; NHS I: female nurses aged 30-55 in 1976; NHS II: female nurses aged 25-42 in 1989; HPFS: male health aged 40-75 professionals in 1986 |
| Criteria exclusion | IBD, PHx CRC, FAP or Lynch syndrome, colonoscopy after index exam for any reason other than surveillance | IBD, hereditary CRC syndrome, bowel resection, missing pathology or colonoscopy report, poor bowel preparation, incomplete exam up to splenic flexure | IBD, PHx CRC or familial hereditary polyposis syndrome | History of colorectal surgery, IBD, incomplete exam (i.e. failed caecal intubation or poor bowel preparation), incomplete baseline questionnaire | Separate for each study | Trial exclusion criteria: IBD, CRC, familial CRC syndrome, bowel resection. Also exclusion criteria pertaining to certain comorbidities and medications. | CRC detected at screening; incomplete exam; inadequate bowel prep; no histological exam or incomplete polyp removal; hereditary CRC syndrome | IBD or CRC prior to, or at, baseline colonoscopy; CRC diagnosis within 1 year of baseline colonoscopy, no documentation of complete examination to cecum with adequate bowel prep | Death or CRC within 6 months of polypectomy |
| Participant description | Values | Values | Values | Values | Values | Values | Values | Values | Values |
| 1. Male sex | 599 (SD 62) | 1501 (54.9%) | 522 (61.9%) | 5166 (66.8%) | 3917 (71.4%) | 62.70% | 89358 (37.8%) | 19893 (96.6%) | 6774 (25%) |
| 2. Age | Mean 57.8 (SD 9.8) | Mean 61 (SD 10) | Median 58 (IQR 52-67) | Mean 51.6 (SD 6.7) | Mean 62.1 (SD 9.43) | Mean 58 (SD 6.7) | Median 56 (IQR 52-60) | Mean 61 (SD 7.6) | Mean 59.9 (SD 9.9) |
| **3. Outcome to be predicted** | | | | | | | | | |
| Outcome | Advanced adenoma or colorectal adenocarcinoma | Advanced colorectal neoplasia at follow-up colonoscopy | Advanced colorectal neoplasia | Metachronous advanced colorectal neoplasia | Metachronous AN within 3-5 years of polypectomy | Advanced colorectal neoplasia | Colorectal cancer incidence and mortality | Metachronous advanced neoplasia | Post polypectomy CRC |
| Outcome definition | AA: adenoma ≥10 mm, HGD, villous histology | CRC; advanced adenoma defined as size ≥10 mm, villous histology (≥75%), HGD (including intramucosal carcinoma or CIS) | Presence of advanced features: ≥10 mm, villous histology, HGD, adenocarcinoma | Invasive cancer and/or advanced adenoma (adenoma ≥1 cm diameter, adenoma with HGD or villous component thereof) | Adenoma ≥1 cm, HGD, tubulovillous or villous histology, adenocarcinoma six months after index colonoscopy | Presence of advanced features: >25% villous histology, high grade dysplasia, adenocarcinoma, ≥1 cm | Histologically confirmed colorectal adenocarcinoma. Incidence (cases per 100,000 person years) | First occurrence of advanced adenoma, incident CRC, fatal CRC 1 or more years after baseline colonoscopy | CRC ≥6 months after polypectomy |
| Time of outcome occurrence | 2nd surveillance colonoscopy | 3 and 5 years | 3 years | 3 and 5 years | 3-5 years of polypectomy | Follow-up surveillance colonoscopy at 3 or 5 years | Follow-up until time of death or end of study (2014) | 1 or mor years after index colonoscopy | Follow up until time of death or end of study (2015) |
| **4. Candidate predictors** | | | | | | | | | |
| Number of candidate predictors (or parameters) assessed | 3 | 7 | 4 | 10 | 11 | 1 | 16 | 17 | 18 |
| Type of predictors | Patient, tumor factors | Patient, tumor factors, surveillance characteristics | Tumor factor | Patient, tumor factors | Patient factors, tumor factors | Tumor factor | Patient factors, tumor factors | Patient, tumor factors and adenoma detection rate | Patient, tumor, procedural factors |
| **5. Sample size** | | | | | | | | | |
| Number of participants | 965 | 2,914 | 843 | 7,730 | 5,483 | 1,948 | 236,089 | 20,598 | 26,741 |
| Number of outcomes/events | 53 | 189 | 229 | 150 | No information | 187 | 439 | No information | 220 |
| Number events per variable (EPV) or per parameter (EPP) | 17.7 | 27 | 57.3 | 15 | Unknown | 187 | 27.4 | Unknown | 12.2 |
| **6. Missing data** | | | | | | | | | |
| Number of participants with any missing value | No information | Up to 20% (no exact figure given) | No information | 361 | Absolute number unclear | 279 | No information | No information | No information on missingness in participants, but most predictors had a missingness of <15% in all cohort |
| Handling of missing data | No information | Other | No information | Complete-case analysis | Other | Complete-case analysis | No information | No information | Other |
| **7. Model development** | | | | | | | | | |
| Modelling method | Logistic regression | Logistic regression | Cox regression | Cox regression | Logistic regression | Logistic regression | Cox regression | Logistic regression | Cox regression |
| **8. Model performance** | | | | | | | | | |
| Calibration measures | Not evaluated | Predicted vs observed ACN risk | Not evaluated | HL test | HL test | Not evaluated | Not evaluated | HL test | Not evaluated |
| Discrimination measures | Not evaluated | C-Statistic | C-Statistic/AUC graph | C-Statistic/Nagelkerke's R2 | C-Statistic/AUC graph | C-Statistic | C-Statistic | C-Statistic/AUC graph | C-Statistic |
| 1. C-statistic | N/A | 0.707 (0.669-0.744) | 0.81 (0.72-0.86) | 0.71 (0.661-0.759) | 0.68 (0.66-0.70) | 0.587 | 0.626 | 0.63 (0.62-0.65) | 0.71 (0.65-0.77) |
| 2. AUC graph | No | No | Yes | No | Yes | Not evaluated | No | Yes | No |
| Overall measures | Model-generated risks of AA in derivation cohort | Not evaluated | Not evaluated | R-squared | Not evaluated | Spec 73.2%; Sens 39.6% | Not evaluated | Not evaluated | Not evaluated |
| Clinical utility | Not evaluated | Not evaluated | Not evaluated | Not evaluated | Not evaluated | Not evaluated | Not evaluated | Net reclassification and colonoscopy use | NRI |
| **9. Model evaluation** | | | | | | | | | |
| Method used for testing model performance | | | | | | | | | |
| 1. Internal validation | None (Apparent performance) | Bootstrap | Cross-validation | Random split data | Random split data | None (Apparent performance) | Bootstrap | Random split data | None (Apparent performance) |
| 2. External validation | Completely independent | None | None | None | None | None | None | None | Completely independent |
| **10. Results** | | | | | | | | | |
| Number of predictors (or parameters) included in final model | 3 | 6 | 3 | 4 | 6 | 1 | 4 | 9 | 9 |
| Final model included predictor weights or regression coefficients | None | Regression coefficients | Regression coefficients | Regression coefficients | None | No information | Regression coefficients | Regression coefficients | Regression coefficients |
| Final model included intercept (or baseline survival) | No | Yes | No | No | No | No information | No | No | No |
| Alternative presentation of the final prediction models | Score chart | Score chart | Score chart | Score chart | None | None | None | On-line calculator | Score system |
| **11. Interpretation** | | | | | | | | | |
| Interpretation of presented model | Model determined risk scoring system applied to development dataset which changed the proportion of individuals into the low- and high-risk categories, respectively. Risks using the model were more extreme than the actual proportions of AAs vs non-AA in the dataset and more individuals were categorized as 'low risk' by the model. The authors suggest that more extreme predicted risks will aid decision-making by clinicians. | Note that both bootstrapping and cross-validation was used. Also multiple models made and compared: full model, simplified score model, US/UK/Dutch guideline models. | Adenoma bulk had comparative predictive characteristics (C-statistic, sensitivity, specificity) to USMSTF guidelines. | The authors report that the number of colonoscopies needed to detect one metachronous ACRN at the 3 year surveillance decreased by 24%. This was surmised from the finding that observed metaochronous CAN prevalence at 3 years was higher in the high MAPS risk group compared to conventional high risk CRN group. | Prespecified 2 cut-off points aiming for 10% increase in sensitivity + specificity compared to USMSTF guidelines. Demonstrated that the model can modestly improve specificity with no deterioration in sensitivity for post-polypectomy risk stratification. | Adenoma bulk had comparative predictive characteristics (C-statistic, sensitivity, specificity) to USMSTF guidelines. | New risk stratification method reduces the number of individuals classified as high risk (who require intensive surveillance) by three-fold compared to current guidelines without increasing risk of CRC in patients with adenoma. Regression coefficients provided by no baseline hazard. | Combined patient, tumor and ADR for multivariable risk model. Did not assess calibration in the validation dataset. Predictors chosen by univariate selection, multivariate backwards elimination, LASSO and BMA. | Created and externally validated postpolypectomy CRC risk model that performed better than the USMSTF guidelines for colonoscopy surveillance in a hospital-based cohort. |
